# The Potential Effects of Human Milk on Morbidity in Very-Low-Birth-Weight Preterm Infants

**DOI:** 10.3390/nu12061882

**Published:** 2020-06-24

**Authors:** Paola Roggero, Nadia Liotto, Orsola Amato, Fabio Mosca

**Affiliations:** 1 Neonatal Intensive Care Unit, Fondazione IRCCS Ca’ Granda Ospedale Maggiore Policlinico, 20122 Milan, Italy; nadia.liotto@policlinico.mi.it (N.L.); orsola.amato@mangiagalli.it (O.A.); fabio.mosca@unimi.it (F.M.); 2 Department of Clinical Sciences and Community Health, University of Milan, 20122 Milan, Italy

Improvements in quality of care have led to a significant reduction in mortality and morbidity in preterm infants, especially very-low-birth-weight (VLBW) infants. Consequently, more efforts are increasingly being made to improve their long-term health.

A central factor of long-term health is nutritional care, and human milk is considered essential in reducing the incidence of comorbidities and improving long-term outcomes. Indeed, it is well known that human milk is the optimal food source for newborns, primarily preterm infants, and that donor human milk is the best alternative when the mother’s milk is not available or is insufficient [1,2,3].

## 1. Human Milk and Comorbidities

Several observational studies have shown that human milk is superior to formula milk in reducing the rate of comorbidities. It is difficult to determine the direct role of human milk in modulating the risk of comorbidities, as randomized controlled trials cannot be conducted due to the ethical implications inherent in the randomization of human milk feeding; thus, observational studies are useful in situations in which randomized controlled trials are unethical [4]. Despite the potential limitations of observational study designs, several studies have shown that human milk feeding can reduce the rate of bronchopulmonary dysplasia (BPD), sepsis, necrotizing enterocolitis (NEC) and severe retinopathy of prematurity (ROP) more than formula feeding can [5,6,7,8,9,10]. In particular, in a multicentre cohort study that included 1433 VLBW infants, Spiegler et al. found that exclusive formula feeding was associated with an increased risk of BPD [odds ratio (OR) = 2.6, NEC: OR = 12.6 and ROP: OR = 1.80] compared to exclusive human milk feeding [11]. In a large monocentric study designed as an interrupted time series study, Alshaikh et al. [12] showed that a human milk quality improvement initiative to implement the rate of human milk in the neonatal intensive care unit was associated with a lower risk of developing NEC (OR = 0.32; 95% confidence interval 0.11–0.93). Similar results were found in a recent study conducted by Cañizo Vázquez et al. [13], in which infants who received donor human milk had a decreased incidence of NEC compared to formula-fed infants (9.1% vs. 3.4%, *p* = 0.055), especially among the group of infants born at a gestational age between 28 and 32 weeks (5.4 vs. 0.0%, *p* = 0.044).

Considering the ethical issues related to the randomization of mothers’ milk, randomized controlled trials studies can be designed to compare the only effects of donor human milk and those of formula milk. Cristofalo et al. showed that the use of donor human milk to supplement the mother’s milk led to a shorter duration of parenteral nutrition and a lower rate of NEC than did formula feeding [14].

A recent meta-analysis that included randomized controlled trials, including a total of 1879 preterm or low-birth-weight infants, demonstrated that formula feeding leads to a higher risk of NEC (risk ratio= 1.87, 95% confidence interval: 1.23–2.85) than does donor human milk feeding [15].

There is evidence regarding the beneficial effect of mother milk on neurodevelopment of preterm infants [16,17], whereas the evidence on beneficial for neurodevelopmental outcome of donor human milk compared to formula milk was moderate [15].

## 2. Duration and Dose of Human Milk and Comorbidities

Another variable important for the evaluation of the possible effect of human milk on morbidity is the intake of human milk and the duration of human milk consumption. Indeed, the available studies showed different designs in terms of the dose and duration of the intervention. For example, Corpeleijn et al. [18] randomly assigned 373 VLBW infants to receive preterm formula or donor human milk for the first 10 days of life. They found similar results between the two groups in terms of the occurrence of sepsis or meningitis, NEC, and death during the first 60 days of life. However, the limitation of this study was that the intervention period was too short, as the authors did not consider the mode of feeding after the first 10 days of life.

Meinzen-Derr et al. conducted a large study and aimed to determine the association between the dose of human milk consumed and the risk of NEC or death among 1272 extremely-low-birth-weight infants. The authors demonstrated that there is a dose-dependent effect of human milk feeding on the risk of NEC or death after the first 2 weeks of life. Specifically, the risk of NEC or death decreased by 13% for every 100 mL/kg of human milk consumed in the first 14 days of life [19]. Moreover, in a systematic review conducted in 2017, the dose-dependent effect of human milk on comorbidities in premature infants was shown. Specifically, the authors revealed that at least 50% of the liquid consumed needs to be human milk to reduce the incidence of NEC [5].

In 2018, Miller et al. conducted a very comprehensive systematic review and meta-analysis of the studies published after 1990 to summarize the available data regarding the mode and dose of feeding and the incidence of comorbidities (NEC, late onset sepsis, BPD, ROP) in preterm infants born before a gestational age of 28 weeks. Specifically, they compared the effects of different modes of feeding, such as exclusive human milk versus exclusive preterm formula (EPTF), any human milk versus EPTF, a higher versus a lower dose of human milk, and unpasteurized versus pasteurized human milk, on the rate of preterm morbidities. The authors concluded that there is evidence of a clear protective effect of a high dose of human milk against NEC and a possible reduction in the risk of developing late onset sepsis and severe ROP in exclusively human milk-fed infants. Compared to unpasteurized human milk, pasteurized human milk did not show any negative effects on preterm comorbidities. This extensive systematic analysis focused on how the mode of feeding in premature infants can have a protective effect against NEC in particular, whose pathogenesis is still unknown. The authors suggest that the higher the human milk intake is, the lower the occurrence rate of NEC. However, the authors conclude that feeding even small doses of human milk is better than exclusive formula feeding [20]. 

In practice, the available data demonstrate that human milk feeding plays a key role in improving the clinical outcomes of preterm infants; nevertheless, the existing studies have some limitations, mainly related to the study design.

In conclusion, a multidisciplinary approach is crucial for preventing the occurrence of comorbidities and improving the clinical outcomes of preterm infants. The best strategy for preterm infants requiring a high level of care should include not only a neonatologist, but also a nutritionist that can design a personalized nutritional intervention.

## References

[1] WHO/UNICEF (2003). Global Strategy for Infant and Young Child Feeding.

[2] Arslanoglu S., Corpeleijn W., Moro G., Braegger C., Campoy C., Colomb V., Decsi T., Domellof M., Fewtrell M., Hojsak I. (2013). Donor human milk for preterm infants: Current evidence and research directions. J. Pediatr. Gastroenterol. Nutr..

[3] Haiden N., Ziegler E.E. (2016). Human Milk Banking. Ann. Nutr. Metab..

[4] Brown J.V.E., Walsh V., McGuire W. (2019). Formula versus maternal breast milk for feeding preterm or low birth weight infants. Cochrane Database Syst. Rev..

[5] Bishop C.E., Vasquez M.M., Petershack J.A., Blanco C.L. (2010). Pasteurized donor human milk for VLBW infants: The effect on necrotizing enterocolitis and related factors. J. Neonatal-Perinat. Med..

[6] Assad M., Elliott M.J., Abraham J.H. (2016). Decreased cost and improved feeding tolerance in VLBW infants fed an exclusive human milk diet. J. Perinatol..

[7] Cacho N.T., Parker L.A., Neu J. (2017). Necrotizing Enterocolitis and Human Milk Feeding A Systematic Review. Clin. Perinatol..

[8] Chowning R., Radmacher P., Lewis S., Serke L., Pettit N., Adamkin D.H. (2016). A retrospective analysis of the effect of human milk on prevention of necrotizing enterocolitis and postnatal growth. J. Perinatol..

[9] Colacci M., Murthy K., Deregnier R.A.O., Khan J.Y., Robinson D.T. (2017). Growth and development in extremely low birth weight infants after the introduction of exclusive human milk feedings. Am. J. Perinatol..

[10] Giuliani F., Prandi G., Coscia A., Cresi F., Di Nicola P., Raia M., Sabatino G., Occhi L., Bertino E. (2012). Donor human milk versus mother’s own milk in preterm VLBWIs: A case control study. J. Biol. Regul. Homeost. Agents.

[11] Spiegler J., Preuß M., Gebauer C., Bendiks M., Herting E., Göpel W., Network G.N. (2016). Does breastmilk influence the development of bronchopulmonary dysplasia?. J. Pediatr..

[12] Alshaikh B., Kostecky L., Blachly N., Yee W. (2015). Effect of a Quality Improvement Project to Use Exclusive Mother’s Own Milk on Rate of Necrotizing Enterocolitis in Preterm Infants. Breastfeed Med..

[13] Cañizo Vázquez D., Salas García S., Izquierdo Renau M., Iglesias-Platas I. (2019). Availability of Donor Milk for Very Preterm Infants Decreased the Risk of Necrotizing Enterocolitis Without Adversely Impacting Growth or Rates of Breastfeeding. Nutrients.

[14] Cristofalo E.A., Schanler R.J., Blanco C.L., Sullivan S., Trawoeger R., Kiechl-Kohlendorfer U., Dudell G., Rechtman D.J., Lee M.L., Lucas A. (2013). Randomized Trial of Exclusive Human Milk Versus Preterm Formula Diets in Extremely Premature Infants. J. Pediatr..

[15] Quigley M., Embleton N.D., McGuire W. (2019). Formula versus donor breast milk for feeding preterm or low birth weight infants. Cochrane Database Syst. Rev..

[16] Eidelman A.I., Feldman R. (2004). Positive effect of human milk on neurobehavioral and cognitive development of premature infants. Adv. Exp. Med. Biol..

[17] Brown Belfort M. (2017). The Science of Breastfeeding and Brain Development. Breastfeed Med..

[18] Corpeleijn W.E., de Waard M., Christmann V., van Goudoever J.B., Jansen-van der Weide M.C., Kooi E.M., Koper J.F., Kouwenhoven S.M., Lafeber H.N., Mank E. (2016). Effect of Donor Milk on Severe Infections and Mortality in Very Low-Birth-Weight Infants: The Early Nutrition Study Randomized Clinical Trial. JAMA Pediatr..

[19] Meinzen-Derr J., Poindexter B., Wrage L., Morrow A.L., Stoll B. (2009). Role of human milk in extremely low birth weight infants’ risk of necrotizing enterocolitis or death. J. Perinatol..

[20] Miller J., Tonkin E., Damarell R.A., McPhee A.J., Suganuma M., Suganuma H., Middleton P.F., Makrides M., Collins C.T. (2018). A Systematic Review and Meta-Analysis of Human Milk Feeding and Morbidity in Very Low Birth Weight Infants. Nutrients.

